# Supplementary material for: Time-Resolved Laurdan Fluorescence Reveals Insights into Membrane Viscosity and Hydration Levels
Source: Biophys J. 2018 Sep 6;115(8):1498–508. doi: 10.1016/j.bpj.2018.08.041 (PMC6257870; doi:10.1016/j.bpj.2018.08.041)
Supplement: Document S1. Supporting Materials and Methods and Figs. S1–S3 [file mmc1.pdf]

Biophysical Journal, Volume *115*

Supplemental Information

Time-Resolved Laurdan Fluorescence Reveals Insights into Membrane Viscosity and Hydration Levels

Yuanqing Ma, Aleš Benda, Joanna Kwiatek, Dylan M. Owen, and Katharina Gaus

Time-resolved Laurdan fluorescence reveals insights into membrane viscosity and hydration levels

Yuanqing Ma, Aleš Benda, Joanna Kwiatek, Dylan M. Owen, and Katharina Gaus

Supplementary information

Comparison of time-resolved GP parameters to TRES measurements

In the time-resolved GP method, each of the extracted parameters represents a corresponding parameter in TRES. For instance, the GP FC value represents *ν(0)* of Laurdan at Franck-Condon state prior to solvent relaxation. Note that due to the limited time resolution of the instrument, it was experimentally difficult to capture the absolute *ν(0)* of Laurdan's Franck-Condon state. It is likely that the fast solvent relaxation at picosecond to sub-nanosecond time scale was not captured in current study. In other words, the estimated GP value of the Laurdan Frank Condon state was already somewhat relaxed in situations where fast solvent relaxation occurs. Instead, as shown in **Fig. 1e-f**, we estimated the of the GP value of apparent Franck-Condon state (GP FC) from the amplitude and offset of the fitted exponential functions. For our data, a double exponential decay fit was required. The GP FR represents the *ν(∞)* in TRES where the solvent relaxation was fully completed. In our data, GP FR was acquired from the offset of the time resolved GP curve fitting. The difference in total amplitudes of the two respective exponential function $\Delta GP = \sum F_i$, represents the extent of solvent relaxation, known as $\Delta\nu$ as in TRES. The kinetics of solvent relaxation known as τ_r in TRES is represented by the intensity averaged decay constant of the time-resolved GP curve, referred to as Ave GPτ here. The time-resolved GP decay curve was tail fitted to double exponential decay function in the lifetime range of 0.3 ns to 20 ns (**Fig. 1e-f**). Finally, the conventional steady state GP was calculated by summing up collected photons in the blue and green spectral windows, respectively, and is called GP SS in the current study. GP SS is predominantly determined by the amounts of photons transferred from blue to green channel in the given lifetime of excited Laurdan molecule. It is partially influenced by the Franck-Condon spectrum and proportion of excited molecules that manage to relax fast enough before they emit the photon.

Supplementary Figures:

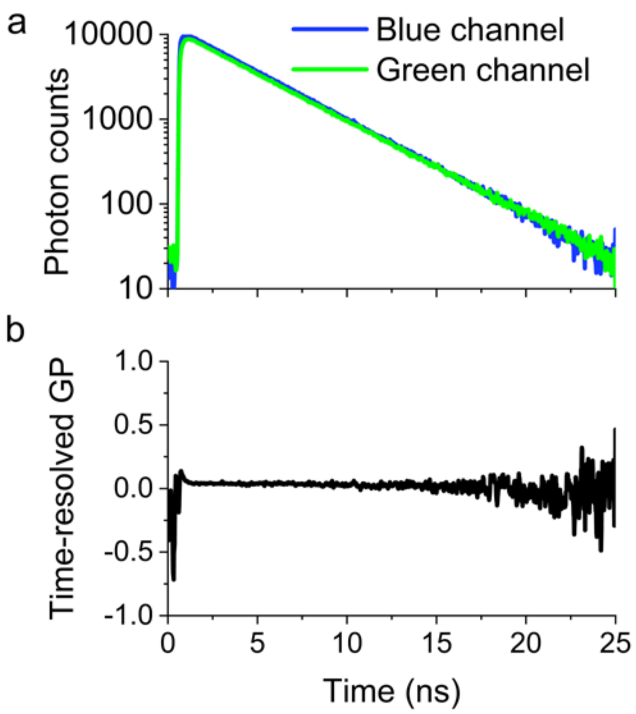

Supplementary Figure 1. Time resolved GP plot of ATTO425. (**a**, **b**) The lifetime decay of ATTO425 in the blue and green channel acquired under the same setting as imaging Laurdan in lipid vesicles (**a**), and the time-resolved GP plot of ATTO425 (**b**). Note that in absence of solvent relaxation, the time resolved GP become a straight line.

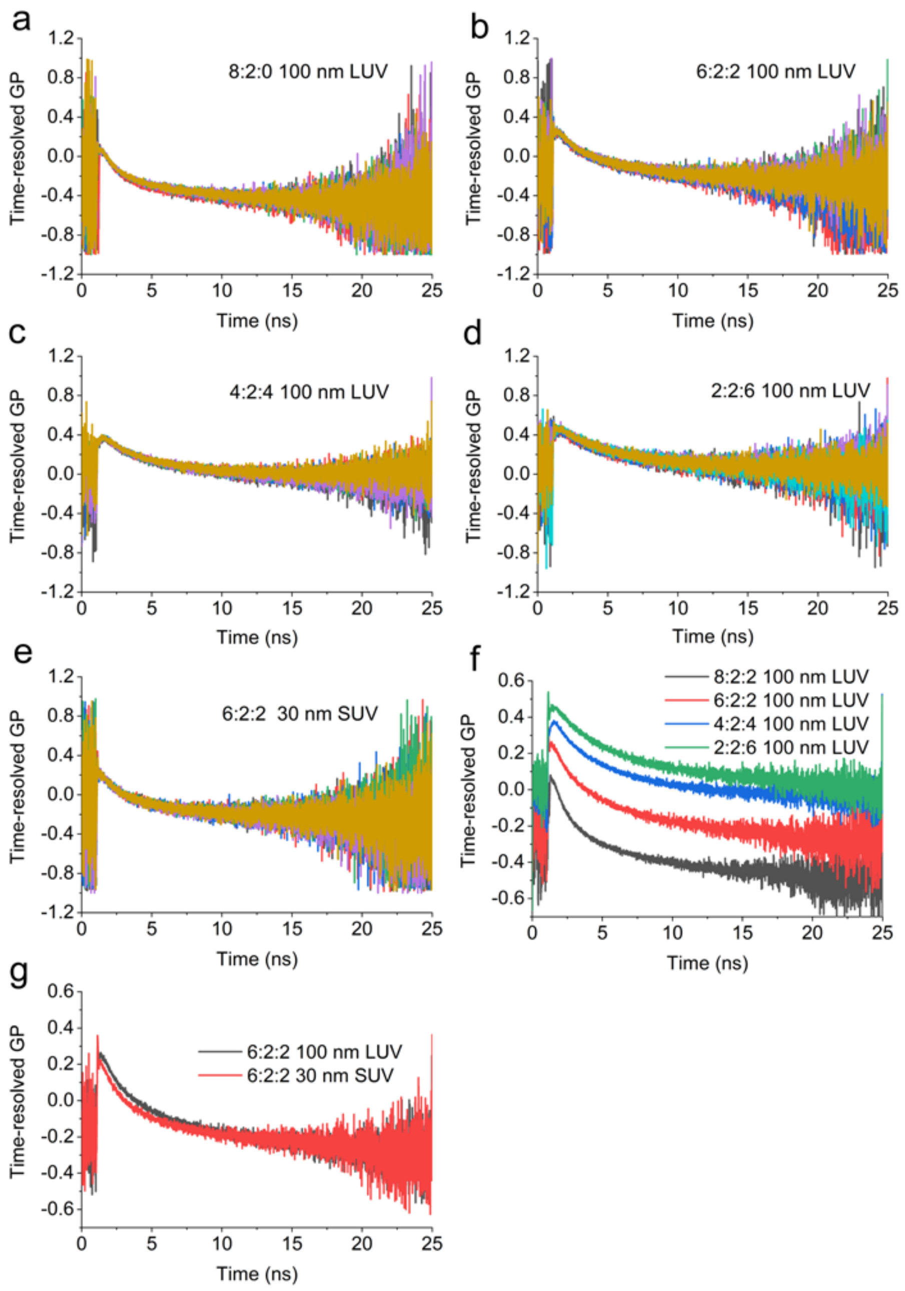

Supplementary Figure 2. Time-resolved GP curves of model membranes with different cholesterol levels and diameter. (**a-e**) Time-resolved GP curves of model membranes with the indicated diameter and ratio of DOPC/sphingomyelin/cholesterol. (**f, g**) Averaged time-resolved GP curves of data shown in a-e (**f**) or b and e (**g**).

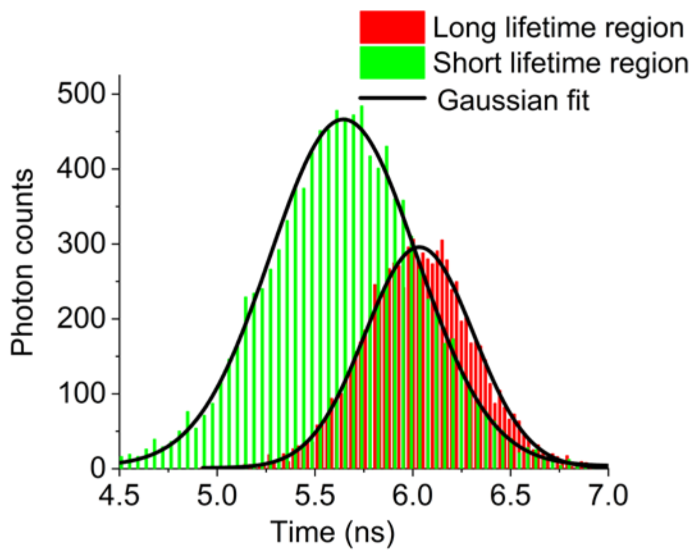

Supplementary Figure 3. Pixel lifetime histograms of the short and long Laurdan lifetime regions of the plasma membrane collected with a SAF objective and shown in Fig. 4b middle and bottom, respectively. The histograms were fitted to single Gaussian distribution functions that peaked at 5.65 ns and 6.03 ns, respectively, and had overall fit of R^2 = 0.995 and R^2 = 0.991 for short and long lifetime regions, respectively.
