# Supplementary material for: Time-Resolved Laurdan Fluorescence Reveals Insights into Membrane Viscosity and Hydration Levels
Source: Biophys J. 2018 Sep 6;115(8):1498–508. doi: 10.1016/j.bpj.2018.08.041 (PMC6257870; doi:10.1016/j.bpj.2018.08.041)
Supplement: Document S2. Article plus Supporting Material [file mmc2.pdf]

Biophysical Journal
Article

Biophysical Society

Time-Resolved Laurdan Fluorescence Reveals Insights into Membrane Viscosity and Hydration Levels

Yuanqing Ma,[1,2] Aleš Benda,[3] Joanna Kwiatek,[1,2] Dylan M. Owen,[4] and Katharina Gaus[1,2,*]
[1]EMBL Australia Node in Single Molecule Science, School of Medical Sciences, [2]ARC Centre of Excellence in Advanced Molecular Imaging, and [3]Biomedical Imaging Facility, Lowy Cancer Research Centre, University of New South Wales, Sydney, New South Wales, Australia; and [4]Department of Physics and Randall Division of Cell and Molecular Biophysics, King's College London, London, United Kingdom

ABSTRACT Membrane viscosity and hydration levels characterize the biophysical properties of biological membranes and are reflected in the rate and extent of solvent relaxation, respectively, of environmentally sensitive fluorophores such as Laurdan. Here, we first developed a method for a time-resolved general polarization (GP) analysis with fluorescence-lifetime imaging microscopy that captures both the extent and rate of Laurdan solvent relaxation. We then conducted time-resolved GP measurements with Laurdan-stained model membranes and cell membranes. These measurements revealed that cholesterol levels in lipid vesicles altered membrane hydration and viscosity, whereas curvature had little effect on either parameter. We also applied the method to the plasma membrane of live cells using a supercritical angle fluorescence objective, to our knowledge the first time fluorescence-lifetime imaging microscopy images were generated with supercritical angle fluorescence. Here, we found that local variations in membrane cholesterol most likely account for the heterogeneity of Laurdan lifetime in plasma membrane. In conclusion, time-resolved GP measurements provide additional insights into the biophysical properties of membranes.

INTRODUCTION

It is now recognized that the plasma membrane of mammalian cells is not simply a homogenous lipid bilayer but is diverse in composition, organization, and shape, giving rise to distinct membrane domains (1). Several membrane models have been proposed to account for the inhomogeneous nature of the plasma membrane such as the lipid raft and picket fence models. The lipid raft hypothesis, for example, suggests that densely packed lipid domains exist within the plasma membrane that are enriched in cholesterol and sphingomyelin and have different biophysical properties to the rest of the membrane (2). Highly sensitive fluorescence techniques such as fluorescence correlation spectroscopy coupled to stimulated emission depletion have successfully revealed the heterogeneous diffusion of raft lipids such as sphingomyelin (3). The picket fence model proposes that the plasma membrane is compartmentalized by a cortical actin network that can temporarily trap membrane proteins (4). Single-particle tracking in intact cells has provided evidence for the picket fence model by analyzing the diffusion trajectory of membrane lipids and proteins (5). However, other membrane properties such as membrane curvature or protein clustering could potentially generate similar anomalous diffusion of membrane molecules (6,7). Thus, methods that provide insights into the biophysical causes of membrane heterogeneity are highly desirable (8).

Polarity-sensitive dyes change their fluorescence properties according to their lipid environments. We and others have previously visualized the lipid-packing properties of cell and model membranes using the polarity-sensitive dye 2-dimethylamino-6-lauroylnaphthalene (Laurdan) (9–11). The main limitation of this approach is the resolution limit of optical microscopy, meaning that membrane domains below ~250 nm cannot be resolved. This limitation can be overcome by using polarity-sensitive dyes in conjunction with super-resolution fluorescence microscopy. This has enabled hydrophobicity mapping with Nile Red (12) and stimulated emission depletion imaging with di-4-ANEPPDHQ, di-4-AN(F)EPPTEA, and NR12S (13). An alternative approach is to use other fluorescence properties of polarity-sensitive dyes (14). For example, we previously

Submitted October 26, 2017, and accepted for publication August 27, 2018.
*Correspondence: k.gaus@unsw.edu.au
Aleš Benda's present address is Imaging Methods Core Facility, BIOCEV, Faculty of Sciences, Charles University, Vestec 252 50, Czech Republic.
Editor: Tobias Baumgart.
https://doi.org/10.1016/j.bpj.2018.08.041
© 2018 Biophysical Society.
This is an open access article under the CC BY license (http://creativecommons.org/licenses/by/4.0/).

used the fluorescence spectra of NR12S (14) to provide evidence that the plasma membrane is better described as a mixture of ordered and disordered lipid phases than as a homogeneous, intermediate environment. Here, we explore whether it is possible to derive a biophysical "signature" from time-resolved measurements of Laurdan fluorescence emission to gain insights into how membrane curvature and local cholesterol concentrations contribute to membrane heterogeneity.

Laurdan is a synthetic, environmentally sensitive dye that is essentially nonfluorescent in water. When incorporated into a lipid bilayer, the functional group of Laurdan is physically located at the carbonyl region of the bilayer (15). However, Laurdan is amphiphilic and can insert into lipid bilayers at different depths and orientations (16,17). In membranes, Laurdan is fluorescent with at least two excited states: the locally excited state, which is intrinsic to the fluorophore, and an internal charge transfer state created by a larger dipole moment. The latter causes the reorientation of the surrounding water molecules to align with the Laurdan dipole moment (18). This process consumes the energy of excited Laurdan molecules so that the frequency of the emitted photons is decreased, which causes a red shift in the emission wavelength. This process is referred to as solvent relaxation (19). Depending on the number of the surrounding water molecules, Laurdan displays a varying degree of solvent relaxation that can be used to describe the lipid environment in membranes (19,20). For instance, in model membranes with distinct but coexisting liquid disordered (Ld) and liquid ordered (Lo) phases, spectroscopic measurements of Laurdan can distinguish the two phases because of their difference in solvent relaxation (19,20).

For a spectral analysis of Laurdan, fluorescence is typically collected in a "blue" and "green" channel, and the normalized difference between the two spectral channels is known as the general polarization (GP) (19,21). Steady-state GP measurements can provide useful estimates of the overall lipid environment (9,22), but for more detailed insights, it is necessary to capture the kinetics of solvent relaxation as well as the fluorescent lifetime of Laurdan. This is because the steady-state GP value of Laurdan is determined by the amount of solvent relaxation that occurs while Laurdan is in its excited state. This situation is similar to the Perrin equation of steady-state anisotropy (23), which can be used to calculate the rotational diffusion of molecules in solution. When the fluorescence lifetime is substantially shorter than the solvent-relaxation process, the steady-state GP approaches the initial GP value as if solvent relaxation has not occurred. When the solvent-relaxation process is much shorter than the Laurdan lifetime, the steady-state GP approaches the fully solvent-relaxed state.

The local membrane environment influences Laurdan lifetime. For instance, Laurdan is sensitive to collisional quenching by water molecules within the membrane through excited-state chromophore-water interactions (10). Increasing the membrane water content by either increasing membrane temperature or decreasing the acyl chain saturation level can lead to a decrease in Laurdan fluorescence lifetime (19,20). The lipid composition and lipid packing impact the local membrane hydration levels, causing a broader distribution in Laurdan lifetime values (15,24–26). Time-resolved spectral measurements such as time-resolved emission spectra (TRES) have been used to capture both the scale and dynamics of Laurdan solvent relaxation (15). With picosecond temporal resolution, TRES can record the entire time course of Laurdan's spectral shift immediately after excitation. From such data, it is possible to calculate the extent of Laurdan solvent relaxation, $\Delta\nu$, defined as the difference between the initial excited state (the so-called Franck-Condon state), $\nu(0)$, and the fully relaxed state, $\nu(\infty)$, using either the overall shift in emission peak or the center of mass of the spectra (15). It has been shown that in model membrane, the extent of Laurdan solvent relaxation, $\Delta\nu$, measured as $\Delta\nu = \nu(0) - \nu(\infty)$, linearly increased with membrane hydration levels (15,26). The rate of solvent relaxation, often expressed as τ, can easily be determined by the rate of the spectral shift. A faster spectral shift corresponds to a faster kinetics of the solvent-relaxation process and vice versa. The speed of solvent relaxation is related to the rotational mobility of the water molecules within the membrane and is often referred to as membrane viscosity (15).

Variations in a dye's local lipid environment, such as an enrichment in saturated lipids or cholesterol and changes in membrane curvature, may alter membrane viscosity and hydration levels and thus potentially the fluorescence properties of Laurdan (10,15,25,27). In this study, we established a time-resolved GP approach as a simplified version of TRES that captures both the scale and kinetics of the Laurdan solvent-relaxation process. We acquired the Laurdan lifetime decay in two spectral windows simultaneously. By taking the ratio of photons as a function of lifetime, the influence of changes in Laurdan lifetime is cancelled out. The time-resolved GP values are only sensitive to the transfer of energy between the two channels, which is proportional to the solvent-relaxation process. From a single fluorescence-lifetime imaging microscopy (FLIM) acquisition, multiple properties of the Laurdan fluorescent emission— including the fluorescence lifetime, the GP value of the apparent Franck-Condon state and the fully solvent-relaxed state, the extent and rate of Laurdan solvent relaxation, and the steady-state GP value—were captured. Using this method, we analyzed Laurdan fluorescence properties for two different types of model membranes, namely, lipid vesicles with different cholesterol concentrations and with different membrane curvatures made of ternary lipid mixtures. We showed that membrane cholesterol caused much more profound changes to Laurdan fluorescence than membrane curvature. We also applied the method to live HeLa

cells, using a supercritical angle fluorescence (SAF) objective to generate FLIM images of the plasma membrane, and found that variations in Laurdan lifetime are likely to be caused by alteration in membrane cholesterol levels in the plasma membrane.

MATERIALS AND METHODS

Preparation of model membranes and cells

Lipid vesicles were prepared from a ternary lipid mixtures containing DOPC (1,2-dioleoyl-snglycero-3-phosphocholine), cholesterol (ovine extract), and sphingomyelin (egg extract; all from Avanti Polar Lipids, Alabaster, AL) at ratios of 8:2:0, 6:2:2, 4:2:4, and 2:2:6 used to represent membranes in Ld phase (8:2:0), membranes with coexisting Ld and Lo phases at low cholesterol concentrations (6:2:2) and at high cholesterol concentrations (4:2:4), and Lo-phase lipid vesicles (2:2:6), respectively. The lipids were mixed in chloroform and Laurdan before lipid extrusion. The mixtures were dried under a nitrogen flow and rehydrated in 10 mM HEPES buffer containing 150 mM NaCl and 100 μM EDTA. This produced a lipid and dye concentration of 1 mM and 5 μM, respectively. For TRES measurement, large multilamellar vesicles in Ld phase were used. For time-resolved GP measurements, large unilamellar vesicles (LUVs) of 100 nm diameter were prepared by extrusion through a 100-nm-sized filter using a lipid extruder (Avanti Polar Lipids), and small unilamellar vesicles (SUVs) of 30 nm diameter were prepared by sonication, respectively. For lipids containing high cholesterol concentrations, the lipid extrusion was performed at 55°C. For SUVs, the sonicated lipids were further purified by centrifugation and filtering through 0.22 μm filters to remove titanium particles from the sonication tip and larger lipid vesicles.

For live-cell imaging with Laurdan, Laurdan in dimethyl sulfoxide was added to cell media to a final concentration of 5 μM and incubated at 37°C at 5% CO_2 incubator for 30 min. Cells were then washed and imaged in Hank's balanced salt solution and monitored for any evidence of phototoxicity or photobleaching during data acquisition.

Spectroscopic measurements and microscopy

TRES measurement was performed with a fluorometer (FluoroMax-4; Horiba, Kyoto Prefecture, Japan). Laurdan-labeled Ld-phase lipid vesicles were excited with a 373 nm photodiode laser pulsed at 20 MHz. Laurdan fluorescence was separated by an emission monochromator and collected with a photon-counting photomultiplier tube. Laurdan lifetime was determined by separating the fluorescence emission into 5 nm bands with a monochromator, scanned from 400 to 580 nm with 20 nm steps. To avoid changes in lifetime due to a loss of emission polarization, the magic angle of 54.7° to the vertical polarization direction was used for both excitation and detection. The excitation laser intensity was tuned so that the detector counting rate was less than 2% of the laser frequency. More than 500,000 photons were collected at each spectral band. The TRES files were exported and analyzed in OriginPro. The instrument response function was deconvoluted from the lifetime decay for each spectral band. The photons collected in the 420–480 and 480–580 nm spectral windows were summed to simulate the experimental conditions for the time-resolved GP measurements, in which photons were separated by a beam splitter at 484 nm.

For the time-resolved GP measurements, a FLIM instrument (Microtime 200; PicoQuant, Berlin, Germany) was used. The microscope body is an inverted Olympus IX71 equipped with a P-733.2CL XY objective scanner (Physik Instrumente, Karlsruhe, Germany). Excitation was realized with a pulsed diode laser (P-C-405 LDH series, 405 nm, at 40 MHz; PicoQuant) so that the laser power at objective was 5 μW. For the time-resolved GP measurements of model membranes, the confocal setup was used, which consisted of a 60×, 1.2 numerical aperture (NA) UPlanSApo water objective (Olympus, Tokyo, Japan) and a 100 μm pinhole. For confocal measurements of cells, the 100×, 1.46 NA PlanApo Oil total internal reflection fluorescence objective (Olympus) was used with a 30 μm pinhole. Fluorescence emission was separated from the reflected excitation laser by an 80/20 transmission/reflection dichroic mirror (21001; CHROMA, Irvine, CA). A 430 nm long-pass filter was inserted to further block the reflected excitation laser. Laurdan fluorescence were collected by two single-photon avalanche diode detectors (PDM series; PicoQuant). A 484 nm long-pass beam splitter was used to split Laurdan fluorescence into 430–484 nm and >485 nm emission bands. It should be noted that the use of a 430 nm long-pass filter meant that some of the fast-emitting blue photons may not have been captured, resulting in a lower ratio of blue/green photons at early time points and lower generalized polarization Fanck-Codon state (GP FC) values. For high-resolution time-resolved GP measurements of the plasma membrane, a 1.0 NA SAF objective (custom made by Dr. Ruckstuhl (28)) was used with the same optical filters and spectral windows. No pinhole was used in the SAF setup as the 100 μm diameter active area of the single-photon avalanche diode detectors acted as pinholes.

The time-resolved Laurdan GP analysis for model and cell membranes was performed with a custom-made LabVIEW program (time-tagged time-resolved data analysis). For lifetime fitting, the photons of each TCSPC channel in the blue and green spectral window were added and replotted as a combined TCSPC histogram. The lifetime decay constant was acquired by fitting the intensity histogram to a single exponential decay function. For the time-resolved GP analysis, the FLIM data of the two spectral windows were loaded simultaneously and time-resolved GP calculated as described in the Results and Discussion. The rising edge (time axis) and offset (amplitude axis) of the decay in the green channel were manually shifted to be aligned to the blue channel to comparable levels and kept constant for all measurements. For time-resolved analysis of the cell plasma membrane, the background pixels were excluded from the analysis by using the intensity threshold combined with a vector representation of the intensity histogram decay, named a phasor plot (29). The phasor approach is a fitting free method that offers higher flexibility and simplicity, such as selection and isolation of pixels displaying specific lifetimes. Because Laurdan fluorescence signals originated from the cell membrane displayed distinctive longer lifetimes than background fluorescence, the pixels containing mostly Laurdan signals can be conveniently selected from the phasor diagram and extracted to produce a cell-membrane-only masked FLIM file. All the subsequent analysis was done on the regenerated background-free masked files. Regions of short and long lifetimes in the plasma membrane were extracted in similar manner.

RESULTS AND DISCUSSION

Time-resolved GP measurements by microscopy

In TRES measurements, Laurdan lifetime is typically recorded at each emission wavelength across the entire emission spectra as shown in Fig. 1, *a* and *b*. The extent and kinetics of solvent relaxation can be extracted from the shift of the emission peak as shown in Fig. 1 *c*. However, data acquisition for TRES measurement is often slow, making it impractical for live-cell imaging. For fast acquisition and imaging of solvent relaxation in live cells, we simplified TRES (Fig. 1, *d–f*) by dividing the spectrum of Laurdan into two spectral windows (blue and green channel) and collected photons in the two channels simultaneously in a time-resolved manner. This allowed us to collect a sufficient amount of data (>200 photons/pixel) for a single cell in ~200 s. Although our approach could not track the shift in the emission peak as done in TRES measurements, the

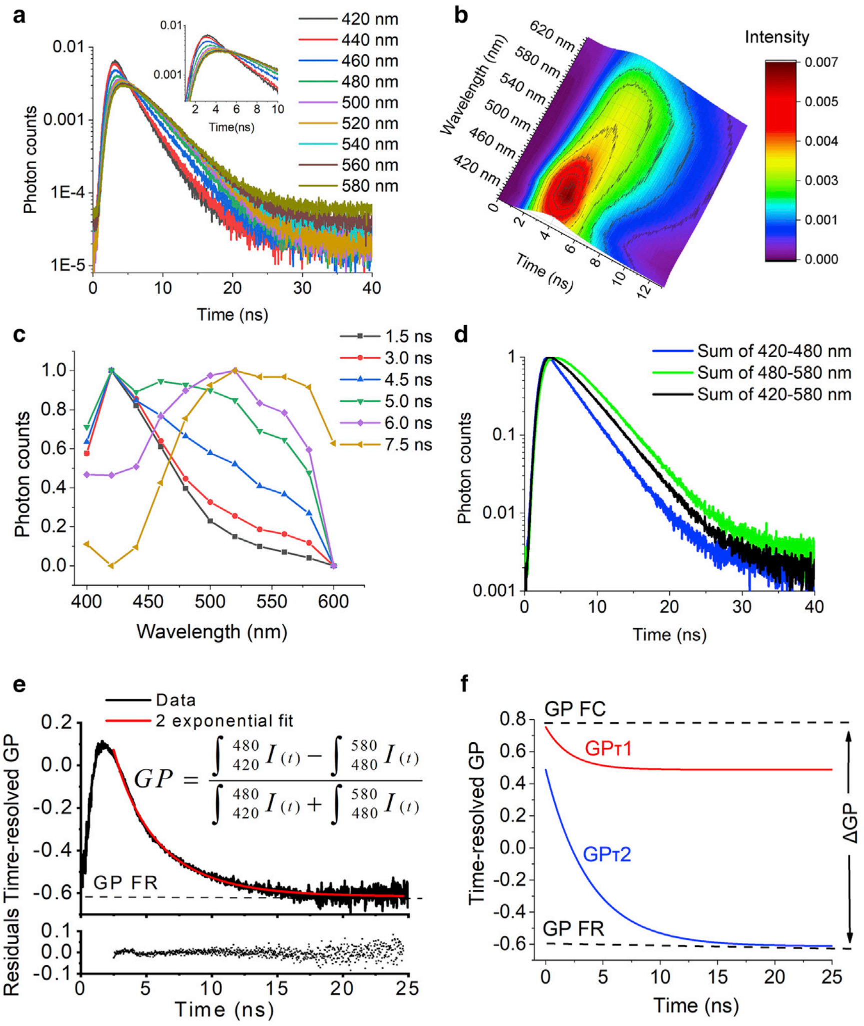

FIGURE 1 Time-resolved spectral analysis of Laurdan solvent relaxation by time-resolved GP measurements. (*a*) The TRES plot collected over 420–580 nm for Laurdan-labeled lipid vesicles in the Ld phase. Lifetime plots are normalized to the area under the curves. The shift in emission peak due to solvent relaxation can be seen in the zoomed region of the peak of the lifetime decay in the inset. (*b*) A three-dimensional surface plot of TRES data shown in (*a*). (*c*) A normalized spectrum of Laurdan at indicated lifetimes from the TRES data shown in (*a*), showing cross-sectional profiles of (*b*) at different lifetimes. Note the red shift in the emission peak at longer lifetimes. (*d*) Laurdan lifetime decays in (*a*) in which photons were grouped into blue (420–480 nm, *blue*), green (480–580 nm, *green*), and total (420–580 nm, *black*) spectral windows by combining photons collected in the corresponding wavelength ranges. (*e*) A time-resolved GP plot of data shown in (*d*). The decay pattern was tail fitted to a double exponential function (*red line*). The distribution of the residuals (*bottom graph*) indicated the appropriateness of fitting. At longer lifetime values, the solvent-relaxation process is completed and the GP value of the fully relaxed state (GP FR, *dotted line*) is estimated from this part of the curve. (*f*) Tail fitting from (*e*) yields two exponential terms as represented by the red and blue single exponential curves. The sum of amplitude of the two decays represents the amount of solvent relaxation from the initial GP FC value to the fully relaxed state GP FR. The apparent Franck-Condon state (GP FC) at time zero is recovered from the sum of ΔGP and GP FR. The fractions and rate constants of the two exponential components GP_{τ_1} and GP_{τ_2} were used to calculate the intensity-averaged rate of solvent-relaxations processes. To see this figure in color, go online.

extent and rate of photon transition from the blue to the green channel contains information on the Laurdan solvent-relaxation process that can be analyzed in a quantitative manner.

As shown in Fig. 1 *d*, the apparent excited-state decay of Laurdan in the blue spectral region is faster compared to the apparent excited-state decay in the green spectral region because of the presence of the solvent relaxation that provides another process, in addition to the standard fluorescence emission, by which the number of molecules emitting in the blue spectral region gets lower with time and thus increases the overall decay rate constant. The apparent excited-state decay in the green spectral region is governed by a mixture of two processes, one being a depopulating process from the Franck-Condon state with a characteristic fluorescence decay profile and the other being a populating process caused by an increase of molecules emitting photons with lower energy due to solvent relaxation. As a result, the apparent excited-state decay constant in the green region is slower than in the blue spectral region. In the absence of solvent relaxation such as for ATTO425 in solution, there is no population redistribution between the blue and the green detection channels at all timescales, and the time-resolved GP plot becomes a straight line (Fig. S1). This result also demonstrates that our method is insensitive to the excited-state fluorescent lifetime of the dye and only records the population redistribution between the two spectral detection channels caused predominately by the solvent-relaxation process. A discussion on the difference between the classical TRES measurements and our measurements can be found in the Supporting Materials and Methods.

We named our approach time-resolved GP. The operation was similar to a time-resolved anisotropy analysis, in which the ratio of photons collected in the horizontal and vertical polarization channels are plotted as a function of lifetime (30). Here, GP is the normalized difference in the number of photons collected in the discrete TCSPC channels of the two spectral channels as a function of time and calculated as

$$\mathrm{GP(t)} = \left(I_{blue}(\mathrm{t})\text{–}I_{green}(\mathrm{t})\right)/\left(I_{blue}(\mathrm{t}) + I_{green}(\mathrm{t})\right).$$

The extent and kinetics of the photon transition can be analyzed from the shape of the plotted curves. Here, time-resolved GP is defined as

$$\text{time} - \text{resolved GP} = GP\ \ FC * \sum F_i * \exp(-t/GP\tau i) + GP\ \ FR,$$

where GP FC represents the initial GP value at time zero immediately after excitation and before the solvent relaxation, known as the Franck-Condon state. Note that because of the limited temporal resolution of the instrument, it was difficult to capture the absolute Franck-Condon state. We thus estimated the apparent Franck-Condon state (GP FC above) from the initial GP value at which the time-resolved GP decay started (Fig. 1, *e* and *f*). This value was recovered by adding the total amplitudes of the exponential decays to the offset of the decay. The offset of the decay was determined by the GP value at which the solvent-relaxation process was completed (i.e., at large values of t), which is referred as GP FR in the above equation and represents the fully solvent-relaxed state. The difference between GP FC and GP FR, named ΔGP, represents the extent of solvent relaxation that has occurred during the measured time window. F_i is the amplitude of the respective exponential decay. ΔGP is calculated from the sum of the amplitudes of the exponential decay terms as $\Delta\mathrm{GP} = \sum F_i$. For our data, a double exponential decay function was sufficient to fit the decay well.

GPτ_i is the respective rate of each solvent-relaxation process. It was determined by tail fitting a double exponential decay function to the time-resolved GP curves in the range of 0.3–20 ns (Fig. 1, *e* and *f*). However, it is known that the Laurdan solvent-relaxation process is a complex process that often requires multiexponential decay fit, and the meaning of each fitted component is not well defined (15,24,25). Therefore, we used the intensity-averaged solvent-relaxation time, referred as Ave GPτ, to estimate the kinetics of the Laurdan solvent-relaxation processes, which is calculated as Ave $\mathrm{GP}\tau = \sum F_i{*}\mathrm{GP}\tau_i^2/\sum F_i{*}\mathrm{GP}\tau_i$. Finally, the conventional steady-state GP was conveniently calculated by summing up all collected photons in the blue and green spectral windows and was called GP SS here.

Time-resolved GP measurements of model membranes with different cholesterol levels

Cholesterol is a rigid and planar sterol that preferentially inserts between saturated phospholipids such as sphingomyelin in lipid bilayers. One of its main properties in biological membranes is to increase lipid packing density. Densely packed membranes are thought to contain fewer water molecules and limit the degree of rotational mobility of lipids. Given the sensitivity of solvent relaxation to membrane hydration and membrane viscosity, it is expected that the membrane cholesterol content could have a distinct impact on the extent versus the rate of solvent relaxation sensed by Laurdan.

We investigated the impact of cholesterol on Laurdan's time-resolved fluorescence with lipid vesicles made from the ternary lipid mixtures of sphingomyelin, DOPC, and varying amount of cholesterol (31,32). Ternary lipid vesicles are generally regarded as better models for complex cellular membranes than single phospholipid membranes. According to the phase diagram for similar ternary lipid vesicles at room temperature (31), we selected four conditions that represent the Ld phase (0% cholesterol), the coexistence of Lo and Ld phases (20 and 40% cholesterol), and the Lo phase (60% cholesterol). Conveniently, the cholesterol concentration increased linearly across the four conditions. The lipid/dye ratio was kept constant at 200:1.

The Laurdan fluorescence of the four LUV preparations with varying amount of cholesterol was collected at room temperature as described in the Materials and Methods. The result showed that lipid vesicles containing higher cholesterol levels displayed a longer overall Laurdan lifetime (Fig. 2 *a*), which implies less quenching from water molecules at the membrane-water interface and thus decreased level of membrane hydration. In agreement, the GP SS values, calculated from the ratio of photons in the blue and green spectral channels, also increased with the membrane cholesterol content (Fig. 2 *b*), suggesting a blue shift in the Laurdan emission spectrum and a decrease in membrane hydration.

The time-resolved GP analysis revealed a more detailed picture of how membrane cholesterol affects the amount and kinetics of solvent relaxation. As in previous TRES studies (15,24), it was insufficient to fit the time-resolved GP curves (Fig. S2) to a single exponential decay function, indicating multiple solvent-relaxation processes with distinctive rate constants take place in these lipid vesicles. We found that Laurdan-labeled ternary lipid mixtures and the plasma membrane could be well fitted to double exponential decay curves. The result showed that increase in cholesterol levels caused an increase in GP FC values (Fig. 2 *c*). It was expected that the GP FC values would be constant, as the true Franck-Condon state of Laurdan is independent from the lipid environment. However, in our measurement, we did not capture all blue photons (see Materials and Methods), so that the amplitude of the fast solvent-relaxation component was reduced. As a consequence, we weighted the apparent GP FC values by the Laurdan lifetime values.

The ΔGP values were substantially larger in vesicles containing lower concentrations of cholesterol (Fig. 2 *e*). Interestingly, the influence of cholesterol on the extent of solvent relaxation was more apparent when cholesterol levels

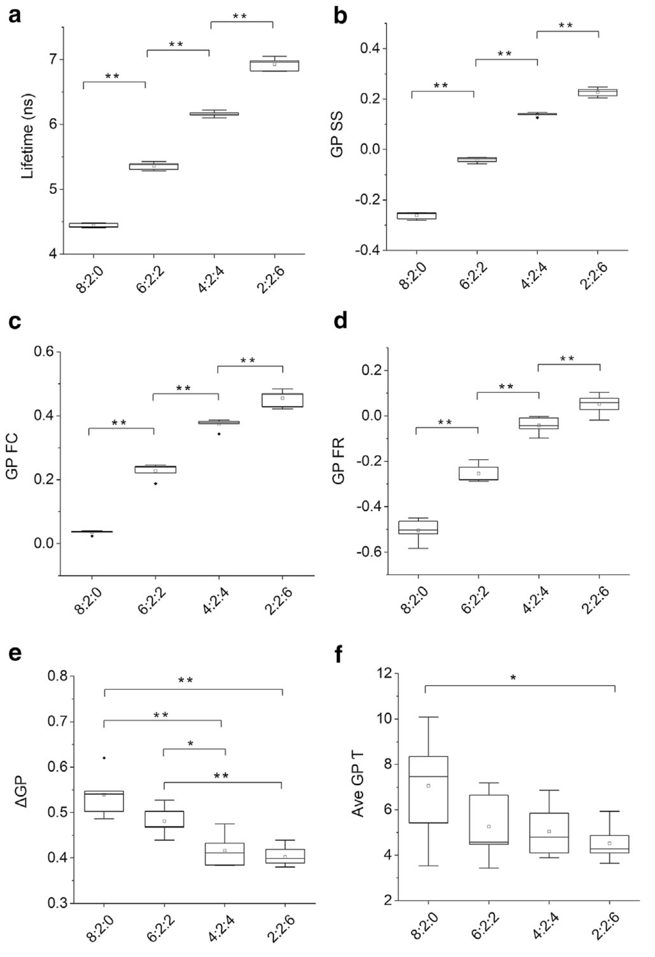

FIGURE 2 Time-resolved GP analysis of model membranes with different cholesterol levels. 100 nm-diameter LUVs of the indicated ratio of DOPC/sphingomyelin/cholesterol were labeled with Laurdan, and Laurdan lifetime (*a*) and time-resolved GP (*b–f*) were measured. Steady-state GP values (GP SS, (*b*)), GP values of the apparent Franck-Condon state (GP FC, (*c*)), fully solvent-relaxed state (GP FR, (*d*)), the extent of solvent relaxation (ΔGP, (*e*)), and the kinetics of the solvent-relaxation processes (Ave GPτ, (*f*)) were derived from photons emitted in the blue (430–484 nm) and green (>485 nm) channels and by fitting the data to a double exponential decay function (as shown in Fig. 1 *f*). Data are for $n = 6$ independent experiments; horizontal bars indicate the median, the upper and lower boundaries of the boxes indicate the 25th and 75th percentiles, vertical bars indicate the 5th and 95th percentiles, filled diamond symbols indicate outliers, and squares indicate mean. One-way ANOVA with Bonferroni post hoc test was used for mean comparison. *$p \leq 0.05$; **$p \leq 0.01$; no asterisk indicates $p > 0.05$.

ranged between 20 and 40%, whereas no significant change in ΔGP values were observed 0–20 and 40–60%. We thus concluded that cholesterol makes the membrane more dehydrated, particularly in the 20–40% range.

As the exact meaning of the individual species of solvent relaxation is not well defined (15,24), we used the intensity-averaged Ave GPτ values to describe the overall Laurdan solvent-relaxation kinetics. Although cholesterol has caused substantial change of Laurdan lifetime decays (Fig. 2 *a*), an increase in cholesterol levels up to 40% did not change the captured kinetics of the Laurdan solvent-relaxations processes but broadened the distribution of the Ave GPτ values. It was also known that the insertion depth of Laurdan and other solvatochromic dyes (16) in the lipid bilayer is not uniform, which could cause a broader distribution of solvent-relaxation processes (15). Further, the gradient of water molecules in the lipid bilayer means that the rotational mobility of water molecules and solvent relaxation are substantially faster at the solvent-lipid interface (24,26). A previous study of Prodan showed that cholesterol could cause the relocation of the dye in the Ld phase of DOPC lipid bilayers (33). A relaxation process related solely to the relocation of the dye molecules in the lipid bilayers would occur on the slow nanosecond timescale (17). Such relocation-initiated solvent relaxation is likely to be responsible for the slow decaying process, visible in the raw time-resolved GP curves (Fig. S2). Following this interpretation, it is likely that an increase in membrane cholesterol reduced the relocation motion of Laurdan in the bilayer. As a result, the distribution of Laurdan solvent-relaxation kinetics became more uniform as observed in Fig. 2 *f*.

The limited temporal resolution of our setup means that a large fraction of the fast solvent-relaxation process was missed. As a result, the calculated Ave GPτ values were more weighted by the slow solvent-relaxation process. An increase in overall Laurdan lifetime via elevated cholesterol levels, for instance, would result in capturing more of the fast solvent-relaxation process and a decrease in Ave GPτ values. Indeed, a significant decrease in Ave GPτ values was observed for vesicles that contained 0–60% of cholesterol. An alternative explanation is that the interactions of charged functional groups of Laurdan with the polar headgroups of nearby phospholipids could produce Laurdan solvent relaxation at slow timescales (24).

Time-resolved GP measurements of model membranes with different curvature

Membrane bending and highly curved membrane structures are often observed in the cells, particularly during endocytosis and exocytosis (34). Membrane curvature directly impacts the spatial packing and rotational flexibility of lipid molecules, which potentially affect membrane hydration and viscosity (25,27,35). In addition, a high local curvature could affect the sorting of lipids into distinctive membrane domains (36). To investigate how Laurdan fluorescence is affected by membrane curvature, we prepared LUVs and SUVs of 100 and 30 nm diameter by lipid extrusion and sonication, respectively. Both types of vesicles had the same lipid composition of DOPC/SM/cholesterol at a ratio of 6:2:2. At room temperature, this lipid mixture produced membranes with coexisting Ld and Lo phases (31,32). The lipid/dye ratio was also kept constant at 200:1. As above, Laurdan was added to the lipid mixture before

vesicle formation so that it could be expected that Laurdan was distributed in both leaflets of the lipid bilayer (25).

Laurdan lifetime was mildly but significantly shortened in the smaller vesicles compared to the larger ones (Fig. 3 *a*). Lifetime values dropped from 5.36 ± 0.06 ns in 100 nm LUVs to 5.12 ± 0.03 ns in 30 nm SUVs. This suggests that an increase in membrane bending caused an increase in the amount of water insertion in the membrane, as one may expect, and this caused a decrease in Laurdan lifetime by water quenching.

The steady-state GP analysis implies membrane solvent relaxation was increased as the GP SS values was reduced from −0.05 ± 0.008 in 100 nm LUVs to −0.08 ± 0.005 in the 30 nm SUVs (Fig. 3 *b*). On the other hand, the time-resolved GP analysis by ΔGP values showed no significant change between 100 nm LUVs and 30 nm SUVs (Fig. 3 *e*), and the GP FR values indicate that the two types of lipid vesicles relaxed to similar levels (Figs. 3 *d* and S2). This implies that the solvent-relaxation processes were similar between the two type of lipid vesicles. We suggest that the decrease in steady-state GP SS value in Fig. 3 *b* was cause by the reduction in Laurdan lifetime. This is because GP SS values were calculated as the ratio of total number of photons transferred from the blue to green channel during the excited Laurdan lifetime. Reduction in lifetime decreases the time window for solvent relaxation to occur so that the GP SS values were also reduced.

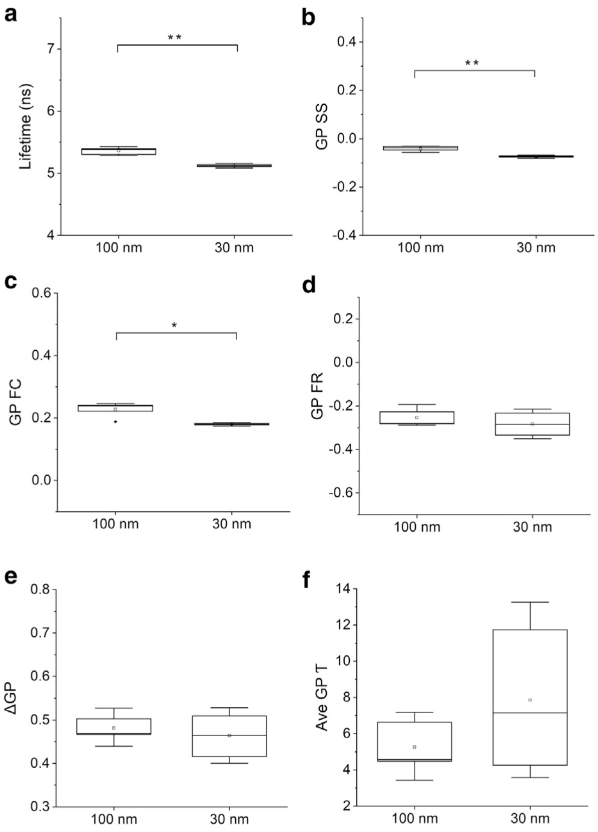

FIGURE 3 Time-resolved GP analysis of lipid vesicles with different curvature. (*a*–*f*) 100 and 30 nm-diameter Laurdan-labeled lipid vesicles were formed from ternary mixtures of DOPC, sphingomyelin and cholesterol at a ratio of 6:2:2, and Laurdan excited-state lifetime (*a*) and time-resolved GP (*b*–*f*) were measured. Steady-state GP values (GP SS, (*b*)), GP values of the Franck-Condon state (GP FC, (*c*)), fully solvent-relaxed state (GP FR, (*d*)), the extent of solvent relaxation (ΔGP, (*e*)), and the kinetics of the solvent-relaxation processes (Ave GPτ, (*f*)) were extracted from fitting time-resolved GP measurements. Data are for $n = 6$ independent experiments. Horizontal bars indicate the median, the upper and lower boundaries of the boxes indicate the 25th and 75th percentiles, vertical bars indicate the 5th and 95th percentiles, filled diamond symbols indicate outliers, and squares indicate mean. Two-sample *t*-test assuming equal variance was used for statistical comparisons. $^*p \leq 0.05$; $^{**}p \leq 0.01$; no asterisk indicates $p > 0.05$.

The rate of solvent relaxation was not influenced by curvature change as shown by Ave GPτ values generated from small and large vesicles (Fig. 3 *f*). This implies that membrane viscosity and the rotational diffusion of membrane water molecules was similar between the 100 nm LUVs and 30 nm SUVs. The distribution of the Ave GPτ values also became more scattered in the 30 nm vesicles. It is likely that as the membrane became more bended, the movement and location of the Laurdan in the bilayer became more frequent, which could increase the amplitude and dynamics of the slow solvent relaxation. In summary, although more water molecules were likely inserted into the lipid bilayer in the highly curved lipid vesicles as suggested by the reduced Laurdan lifetime, our time-resolved GP measurement suggests that the extent of solvent relaxation was not substantially changed. Our interpretation is consistent with a previous analysis of curvature using other solvatochromic dyes that concluded that changes in membrane curvature had no influence on the extent of solvent relaxation and membrane hydration levels, but increased curvature accelerated the rate of solvent-relaxation process at fast subnanosecond timescales (25). Taken together, in our experiments with limited temporal resolution, membrane curvature did not influence the rate or extent of solvent relaxation, suggesting membrane cholesterol and membrane curvature have very different impacts on the time-resolved GP measurements of Laurdan.

Laurdan lifetime imaging of the plasma membrane in live cells with SAF

Finally, we investigated whether time-resolved GP could be performed on cell membranes and whether such measurements provided additional insights as they have done for model membranes. One of the inevitable complications of the cell imaging with Laurdan is that a large fraction of Laurdan molecules become internalized and associated with the internal membranes. The lipid composition of the internal membrane differs substantially from the plasma membrane, resulting in differences in Laurdan fluorescence (10,11,13). As demonstrated in the confocal images of Laurdan-labeled HeLa cells (Fig. 4 *a*), a large fraction of Laurdan was internalized and displayed a shorter lifetime

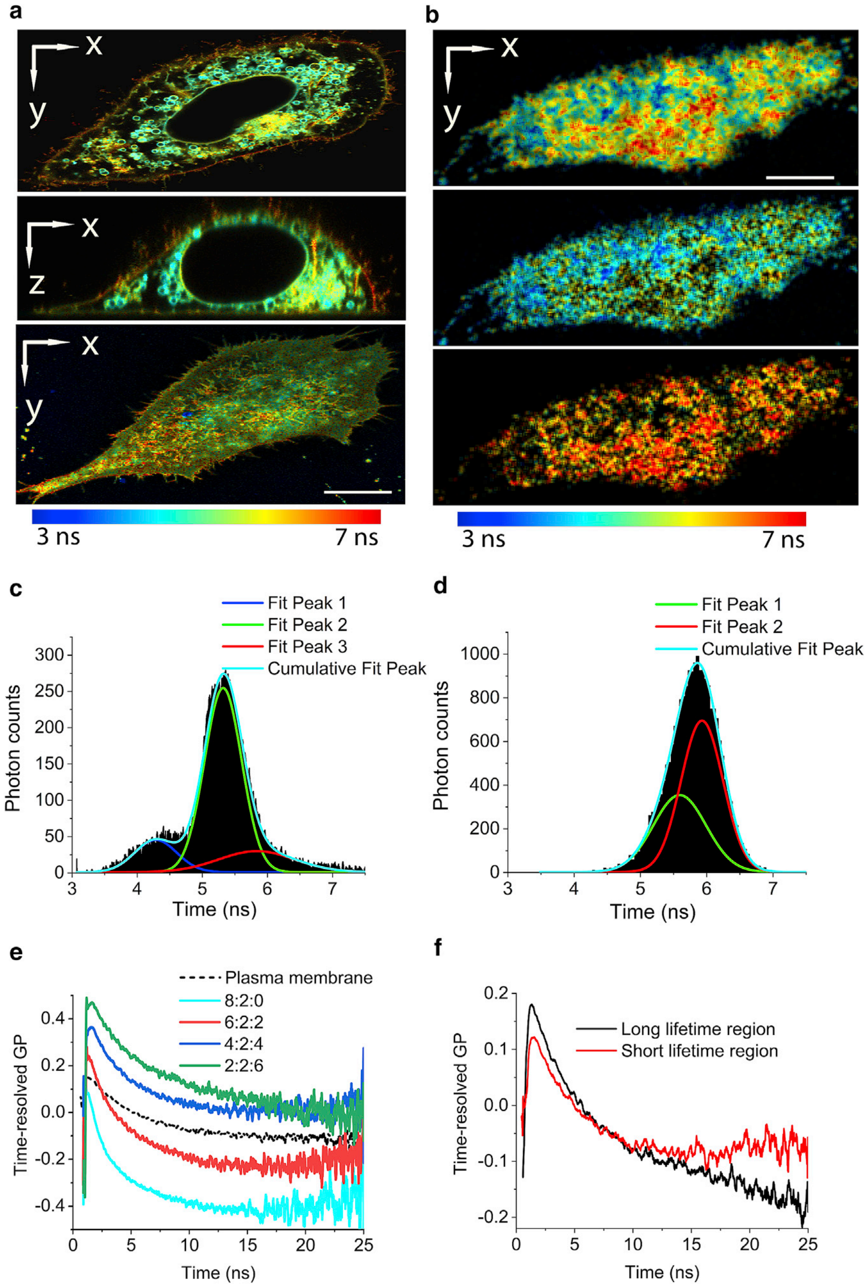

FIGURE 4 Laurdan lifetime imaging and time-resolved GP analysis of the live cell plasma membrane. (*a*) Representative confocal FLIM images of Laurdan-labeled live HeLa cells obtained at the center of the cell (*top*) and adjacent to the glass coverslip (*bottom*) and corresponding *xz* cross section (*middle*). Scale bars, 3 μm; color scale indicates Laurdan fluorescence lifetimes. (*b*) An SAF FLIM image of Laurdan-labeled HeLa cell collected with the same excitation laser setting as in (*a*). Photons from blue and green detectors were grouped (*top image*). Two-mask FLIM files were created by gating photons into shorter (*middle*) and longer (*bottom*) components according to their position on the phasor plot. Images are representative of seven cells. (*c* and *d*) Pixel lifetime histograms of confocal FLIM (*c*) and SAF FLIM (*d*) images, shown in (*a*) and (*b*), respectively. Histograms were fitted (*cyan curve*) to three (*c*) and two (*d*) Gaussian distributions, resulting in an overall fit of $R^2 = 0.978$ (*c*) and $R^2 = 0.951$ (*d*), respectively. (*e*) Time-resolved GP curves of the plasma membrane (*black dotted line*) and model membranes with varying cholesterol levels (from Fig. 2). (*f*) Time-resolved GP curves of the photons collected from short and long Laurdan lifetime regions in the plasma membrane as shown in (*b*) (*middle* and *bottom panel*). To see this figure in color, go online.

compared to the plasma membrane. Even with a 100×, high 1.46 NA objective and a 30 μm pinhole (diameter that is equivalent to approximately one-third of one Airy unit), the confocal image of plasma membrane still contained signals from internal membranes.

To reject Laurdan signals from internal membranes, we employed an SAF objective on a commercial microscope (Microtime 200; PicoQuant) as previously described (37). SAF is a surface-enhanced fluorescence imaging technique in which the near-field energy of the fluorophores located at the glass-water interface is converted into far-field fluorescence because of the surface resonance effect (28). The majority of the fluorescence emitted at supercritical angles (38) can be collected by a parabolic mirror inside the SAF objective. As seen in Fig. 4 *b*, images collected with the SAF objective are reminiscent of total internal reflection fluorescence images. The sample-scanning regime of the microscope allowed us to combine SAF with time-correlated single photon counting (TCSPC) for fast fluorescence spectroscopy analysis (37,39) and lifetime measurement of the plasma membrane, as demonstrated for the first time, to our knowledge, in this study. To make the data collected with the SAF objective comparable to the model membrane data obtained with a confocal setup, the microscope settings

were kept identical to those used in the cholesterol and curvature experiments.

Compared to the FLIM histogram collected with confocal setup that had a broad distribution (Fig. 4 *c*), the Laurdan lifetime histogram distribution from the SAF acquisition was much narrower (Fig. 4 *d*). When the distributions of the pixel lifetime histogram were fitted to multiple Gaussian distributions, the FLIM data from the confocal setup could only be described with three Gaussian functions, which centered at 4.28, 5.32, and 5.83 ns and had a full width at half maxima of 0.64, 0.57, and 1.16 ns, respectively. In contrast, the lifetime histogram of SAF FLIM (Fig. 4 *d*) could be described by two Gaussian functions, which centered at 5.59 and 5.93 ns with full width at half maxima values of 0.79 and 0.63 ns, respectively. The 4.28 ns peak in the confocal FLIM data was completely absent in the SAF acquisition, suggesting these signals originated from Laurdan in intracellular membranes. This demonstrates the advantage of membrane lifetime imaging with SAF.

The lifetimes of the two identified lifetime populations in the SAF FLIM data (5.59 ± 0.51 and 5.93 ± 0.46 ns) were vastly different to the lifetime values of Laurdan in Ld- (4.43 ± 0.03 ns) and Lo- (6.93 ± 0.09 ns) phase model membranes (Fig. 2 *a*). This implies that the plasma membrane may not be a simple mixture of the Lo and Ld phases as observed in the model membranes (11) but does not exclude the possibility of Ld and Lo mixtures below the resolution limit. Comparing the lifetime data of the plasma membrane to model membranes with different cholesterol concentration suggests that the cholesterol composition of plasma membrane lies between 20 and 40%, agreeing with previous studies that report cholesterol concentrations in the plasma membrane of 20–50% (40,41).

It was notable from the FLIM images of the cells that the fluorescence lifetime of Laurdan in plasma membrane was highly heterogeneous (Fig. 4 *b*). There were many structures that displayed longer lifetime values than the remaining sections of the membrane. To gain a better understanding of the difference of lipid environment between the punctate regions, we investigated how the Laurdan solvent-relaxation process differed between those longer and shorter lifetime regions. To do so, we split the data into short- and long-lifetime regions using the phasor approach (29) (see Materials and Methods for details, Fig. 4 *b*). The pixel histogram analysis of the isolated FLIM files showed that each component could be described by a single Gaussian function (Fig. S3). We then performed time-resolved GP analysis for the short and long lifetime regions (as well as total plasma membrane). To keep the analysis consistent with the model membrane experiments, we used a double exponential decay function for fitting. The time-resolved curve of the plasma membrane is shown as a black dotted line in Fig. 4 *e* and compared to the time-resolved GP curves of lipid vesicles with varying cholesterol concentrations as indicated. The time-resolved curves of the long and short lifetime regions are shown in Fig. 4 *f*, and their fitted values in Table 1.

The analysis of the long and short lifetime regions in the plasma membrane suggest that the solvent-relaxations processes between the two membrane regions were substantially different. The longer lifetime components (6.28 ± 0.26 ns) displayed substantially larger amount of solvent relaxation than the short lifetime components (5.42 ± 0.23 ns) as shown by the higher ΔGP values shown in Table 1. This was attributed to the greater amplitudes of the time-resolved GP curves, as shown in Fig. 4 *f*. As a result, the GP FC values increased and GP FR values decreased for the long lifetime regions. We speculate that although both the long- and short-lifetime regions experienced a fast decay in the first ~7 ns, the long-lifetime region was dominated by a slow and continuous decay afterwards, whereas the short-lifetime region flattened out in comparison. It is likely that the increased amplitude of the slow solvent-relaxation process was responsible for the significant increase of Ave GPτ values in the longer lifetime region as shown in Table 1.

Although there was a substantial difference of Laurdan solvent relaxation between the short- and long-lifetime regions of the cell plasma membrane, the exact cause for the change is unclear. The change in the time-resolved GP decays between the short and long lifetime region showing in Fig. 4 *f* resembled the condition of increase of cholesterol levels from 40 to 60% in model membrane. The increase of cholesterol has caused overall upwards shift at early time points and a slow and continuous decay at latter part of the time-resolved GP curves as shown in Fig. 4 *e*. Thus we conclude that variation in local cholesterol levels, rather than changes in membrane curvature, may cause the heterogeneity in the plasma membrane.

TABLE 1 Time-Resolved GP Values for Cell Plasma Membrane Extracted from SAF FLIM Images

Lifetime Region	Overall Lifetime (ns)	GP SS	GP FC	ΔGP	GP FR	Ave GPτ
Total	5.83 ± 0.19	0.066 ± 0.05	0.21 ± 0.19	0.27 ± 0.13	−0.062 ± 0.13	4.81 ± 1.21
Short	5.42 ± 0.23	0.071 ± 0.09	0.19 ± 0.11	0.21 ± 0.11	−0.013 ± 0.11	3.42 ± 1.72
Long	6.28 ± 0.26	0.062 ± 0.07	0.23 ± 0.09	0.34 ± 0.06	−0.116 ± 0.06	6.08 ± 1.54
Significance	*	NS	*	*	*	*

NS, not significant ($p > 0.05$)

*$p \leq 0.05$ paired *t*-test

CONCLUSIONS

In this study, we have developed a fast time-resolved GP analysis method for FLIM data that is capable of extracting multiple parameters from Laurdan solvent relaxation. The time-resolved GP analysis described here quantifies the extent and rate of photon transition from the blue to green channel for a given lifetime of excited Laurdan molecules. Compared to the conventional steady-state GP and standard lifetime measurements, the current time-resolved GP analysis captures some aspects of solvent relaxation but has a limited temporal resolution, which means that the fast solvent-relaxation process and the true Franck-Condon state were not captured.

Investigation of lipid vesicles of varying cholesterol concentration and curvature demonstrated that time-resolved GP provided additional insight into Laurdan solvent relaxation compared to the conventional lifetime and steady-state measurements. It was also concluded that increasing membrane cholesterol levels reduced the extent of solvent relaxation by making the membrane less hydrated. High cholesterol levels are likely to enhance lipid packing so that the local motion of the inserted Laurdan molecules was reduced. In comparison to the effect of cholesterol, membrane curvature had relatively little influence on Laurdan solvent relaxation.

We also investigated whether the Laurdan signal from plasma membrane could be analyzed in a similar manner, utilizing an SAF objective to generate Laurdan FLIM images of the plasma membrane. Our time-resolved GP data is consistent with the plasma membrane having a membrane cholesterol level of 20–40%. Although highly fluid and mobile, the plasma membrane also displayed local heterogeneity, which was reflected in the two distinctive populations in Laurdan lifetimes. The regions with long Laurdan lifetimes were comparable to model membranes containing more than 40% cholesterol, suggesting that the observed membrane heterogeneity was more likely caused by the modulation in membrane cholesterol levels than by changes in membrane curvature. However, it should be taken into account that physical bending of membranes can cause the redistribution of membrane lipids. High membrane curvature can cause an agitation of the local thermodynamic energy, in which lipids of different shapes are redistributed along the curvature to minimize the strain on the membrane (36). Thus, an inhomogeneous lateral distribution of membrane lipids could be linked to variations in membrane curvature (42). For instance, it was shown that highly curved caveolae are also highly enriched in cholesterol and sphingomyelin (43). Another study showed that local cholesterol levels strongly correlated with membrane surface of high curvature such as microvilli and filopodia (42). Therefore, different underlying factors could contribute to the membrane heterogeneity observed with Laurdan, but the simplest explanation is varying local cholesterol concentrations.

There are also technical and biological issues that should be taken into consideration when performing time-resolved GP measurements. For example, the use of a 405 nm laser is not ideal for Laurdan excitation (44) and potentially causes phototoxicity and changes to the physiology of the cell. The use of a 430 nm long-pass filter in the emission path caused the loss of fast-decaying photons in the ultrablue spectral regions. This reduced the amplitude of the fast solvent-relaxation components and prevented us from capturing the true Franck-Condon state of the excited Laurdan. Because Lauran solvent relaxation in lipid membranes occurs via multiple different time-dependent processes (15,25), a relatively large number of photons need to be collected, resulting in the temptation to stain membranes with a high concentration of Laurdan, which might disturb the physiological conditions of the cell. Further, given the membrane permeability of the probe and the rapid internalization of Laurdan in cells, good optical sectioning is required to measure the properties of the plasma membrane. In terms of data interpretation, we attributed the change of time-resolved fluorescence of Laurdan to the lipid environment (45). However, the high concentration of proteins in cell membranes may also affect Laurdan solvent relaxations. We thus conclude that with improvement of the instrument limitations, the time-resolved GP measurement can provide additional insight into the biophysical properties of lipid environments and the heterogeneity of the plasma membrane.

SUPPORTING MATERIAL

Supporting Materials and Methods and three figures are available at http://www.biophysj.org/biophysj/supplemental/S0006-3495(18)31019-1.

AUTHOR CONTRIBUTIONS

Y.M. generated data, analyzed data, and wrote the manuscript. A.B. established analysis. J.K. conducted experiments. D.M.O. contributed to data interpretation and manuscript preparation. K.G. designed the study and wrote the manuscript.

ACKNOWLEDGMENTS

Y.M. and K.G. acknowledge funding from the National Health and Medical Research Council of Australia (APP1139003 to Y.M., APP1059278 to K.G.) and Australia Research Council (CE140100011 to K.G.). A.B. acknowledges the support from CZ.02.1.01/0.0/0.0/16_013/0001775 funded by the Ministry of Education, Youth and Sports, Czech Republic.

REFERENCES

1. Ma, Y., E. Hinde, and K. Gaus. 2015. Nanodomains in biological membranes. *Essays Biochem.* 57:93–107.

2. Simons, K., and E. Ikonen. 1997. Functional rafts in cell membranes. *Nature.* 387:569–572.

3. Eggeling, C., C. Ringemann, ..., S. W. Hell. 2009. Direct observation of the nanoscale dynamics of membrane lipids in a living cell. *Nature.* 457:1159–1162.

4. Kusumi, A., H. Ike, …, T. Fujiwara. 2005. Single-molecule tracking of membrane molecules: plasma membrane compartmentalization and dynamic assembly of raft-philic signaling molecules. *Semin. Immunol.* 17:3–21.
5. Sako, Y., and A. Kusumi. 1994. Compartmentalized structure of the plasma membrane for receptor movements as revealed by a nanometer-level motion analysis. *J. Cell Biol.* 125:1251–1264.
6. Trimble, W. S., and S. Grinstein. 2015. Barriers to the free diffusion of proteins and lipids in the plasma membrane. *J. Cell Biol.* 208:259–271.
7. Weiss, M., H. Hashimoto, and T. Nilsson. 2003. Anomalous protein diffusion in living cells as seen by fluorescence correlation spectroscopy. *Biophys. J.* 84:4043–4052.
8. Carquin, M., L. D'Auria, …, D. Tyteca. 2016. Recent progress on lipid lateral heterogeneity in plasma membranes: from rafts to submicrometric domains. *Prog. Lipid Res.* 62:1–24.
9. Gaus, K., T. Zech, and T. Harder. 2006. Visualizing membrane microdomains by Laurdan 2-photon microscopy. *Mol. Membr. Biol.* 23:41–48.
10. Golfetto, O., E. Hinde, and E. Gratton. 2013. Laurdan fluorescence lifetime discriminates cholesterol content from changes in fluidity in living cell membranes. *Biophys. J.* 104:1238–1247.
11. Owen, D. M., C. Rentero, …, K. Gaus. 2011. Quantitative imaging of membrane lipid order in cells and organisms. *Nat. Protoc.* 7:24–35.
12. Bongiovanni, M. N., J. Godet, …, S. F. Lee. 2016. Multi-dimensional super-resolution imaging enables surface hydrophobicity mapping. *Nat. Commun.* 7:13544.
13. Sezgin, E., F. Schneider, …, C. Eggeling. 2017. Polarity-sensitive probes for superresolution stimulated emission depletion microscopy. *Biophys. J.* 113:1321–1330.
14. Nicovich, P. R., J. M. Kwiatek, …, K. Gaus. 2018. FSCS reveals the complexity of lipid domain dynamics in the plasma membrane of live cells. *Biophys. J.* 114:2855–2864.
15. Jurkiewicz, P., J. Sýkora, …, M. Hof. 2005. Solvent relaxation in phospholipid bilayers: principles and recent applications. *J. Fluoresc.* 15:883–894.
16. Demchenko, A. P., Y. Mély, …, A. S. Klymchenko. 2009. Monitoring biophysical properties of lipid membranes by environment-sensitive fluorescent probes. *Biophys. J.* 96:3461–3470.
17. Demchenko, A. P., and N. V. Shcherbatska. 1985. Nanosecond dynamics of charged fluorescent probes at the polar interface of a membrane phospholipid bilayer. *Biophys. Chem.* 22:131–143.
18. Weber, G., and F. J. Farris. 1979. Synthesis and spectral properties of a hydrophobic fluorescent probe: 6-propionyl-2-(dimethylamino)naphthalene. *Biochemistry.* 18:3075–3078.
19. Parasassi, T., and E. Gratton. 1995. Membrane lipid domains and dynamics as detected by Laurdan fluorescence. *J. Fluoresc.* 5:59–69.
20. Parasassi, T., E. K. Krasnowska, …, E. Gratton. 1998. Laurdan and Prodan as polarity-sensitive fluorescent membrane probes. *J. Fluoresc.* 8:365–373.
21. Parasassi, T., G. De Stasio, …, E. Gratton. 1990. Phase fluctuation in phospholipid membranes revealed by Laurdan fluorescence. *Biophys. J.* 57:1179–1186.
22. Gaus, K., E. Gratton, …, W. Jessup. 2003. Visualizing lipid structure and raft domains in living cells with two-photon microscopy. *Proc. Natl. Acad. Sci. USA.* 100:15554–15559.
23. Van Holde, K. E., C. Johnson, …, P. S. Ho. 1998. Principles of Physical Biochemistry. Prentice Hall, Upper Saddle River, NJ.
24. Sýkora, J., P. Kapusta, …, M. Hof. 2002. On what time scale does solvent relaxation in phospholipid bilayers happen? *Langmuir.* 18:571–574.
25. Sýkora, J., P. Jurkiewicz, …, M. Hof. 2005. Influence of the curvature on the water structure in the headgroup region of phospholipid bilayer studied by the solvent relaxation technique. *Chem. Phys. Lipids.* 135:213–221.
26. Jurkiewicz, P., A. Olzyńska, …, M. Hof. 2006. Headgroup hydration and mobility of DOTAP/DOPC bilayers: a fluorescence solvent relaxation study. *Langmuir.* 22:8741–8749.
27. Zhang, Y. L., J. A. Frangos, and M. Chachisvilis. 2006. Laurdan fluorescence senses mechanical strain in the lipid bilayer membrane. *Biochem. Biophys. Res. Commun.* 347:838–841.
28. Ruckstuhl, T., D. Verdes, …, S. Seeger. 2011. Simultaneous near-field and far-field fluorescence microscopy of single molecules. *Opt. Express.* 19:6836–6844.
29. Digman, M. A., V. R. Caiolfa, …, E. Gratton. 2008. The phasor approach to fluorescence lifetime imaging analysis. *Biophys. J.* 94:L14–L16.
30. Kapusta, P., R. Erdmann, …, M. Wahl. 2003. Time-resolved fluorescence anisotropy measurements made simple. *J. Fluoresc.* 13:179–183.
31. de Almeida, R. F., A. Fedorov, and M. Prieto. 2003. Sphingomyelin/phosphatidylcholine/cholesterol phase diagram: boundaries and composition of lipid rafts. *Biophys. J.* 85:2406–2416.
32. Veatch, S. L., and S. L. Keller. 2005. Miscibility phase diagrams of giant vesicles containing sphingomyelin. *Phys. Rev. Lett.* 94:148101.
33. Bondar, O. P., and E. S. Rowe. 1999. Preferential interactions of fluorescent probe Prodan with cholesterol. *Biophys. J.* 76:956–962.
34. McMahon, H. T., and J. L. Gallop. 2005. Membrane curvature and mechanisms of dynamic cell membrane remodelling. *Nature.* 438:590–596.
35. Lee, J. C. M., R. J. Law, and D. E. Discher. 2001. Bending contributions to hydration of phospholipid and block copolymer membranes: unifying correlations between probe fluorescence and vesicle thermoelasticity. *Langmuir.* 17:3592–3597.
36. Baumgart, T., S. T. Hess, and W. W. Webb. 2003. Imaging coexisting fluid domains in biomembrane models coupling curvature and line tension. *Nature.* 425:821–824.
37. Ma, Y., A. Benda, …, K. Gaus. 2016. Measuring membrane association and protein diffusion within membranes with supercritical angle fluorescence microscopy. *Biomed. Opt. Express.* 7:1561–1576.
38. Emmanuel, F., and G. Samuel. 2008. Surface enhanced fluorescence. *J. Phys. D Appl. Phys.* 41:013001.
39. Ries, J., T. Ruckstuhl, …, P. Schwille. 2008. Supercritical angle fluorescence correlation spectroscopy. *Biophys. J.* 94:221–229.
40. Mitra, K., I. Ubarretxena-Belandia, …, D. M. Engelman. 2004. Modulation of the bilayer thickness of exocytic pathway membranes by membrane proteins rather than cholesterol. *Proc. Natl. Acad. Sci. USA.* 101:4083–4088.
41. van Meer, G., D. R. Voelker, and G. W. Feigenson. 2008. Membrane lipids: where they are and how they behave. *Nat. Rev. Mol. Cell Biol.* 9:112–124.
42. Wüstner, D. 2007. Plasma membrane sterol distribution resembles the surface topography of living cells. *Mol. Biol. Cell.* 18:211–228.
43. Simons, K., and D. Toomre. 2000. Lipid rafts and signal transduction. *Nat. Rev. Mol. Cell Biol.* 1:31–39.
44. Tomin, V., M. Brozis, and J. Heldt. 2003. The red-edge effects in Laurdan solutions. *Zeitschrift für Naturforschung A.* 58:109–117.
45. Dinic, J., H. Biverståhl, …, I. Parmryd. 2011. Laurdan and di-4-ANEPPDHQ do not respond to membrane-inserted peptides and are good probes for lipid packing. *Biochim. Biophys. Acta.* 1808:298–306.

Biophysical Journal, Volume *115*

Supplemental Information

Time-Resolved Laurdan Fluorescence Reveals Insights into Membrane Viscosity and Hydration Levels

Yuanqing Ma, Aleš Benda, Joanna Kwiatek, Dylan M. Owen, and Katharina Gaus

Time-resolved Laurdan fluorescence reveals insights into membrane viscosity and hydration levels

Yuanqing Ma, Aleš Benda, Joanna Kwiatek, Dylan M. Owen, and Katharina Gaus

Supplementary information

Comparison of time-resolved GP parameters to TRES measurements

In the time-resolved GP method, each of the extracted parameters represents a corresponding parameter in TRES. For instance, the GP FC value represents *ν(0)* of Laurdan at Franck-Condon state prior to solvent relaxation. Note that due to the limited time resolution of the instrument, it was experimentally difficult to capture the absolute *ν(0)* of Laurdan's Franck-Condon state. It is likely that the fast solvent relaxation at picosecond to sub-nanosecond time scale was not captured in current study. In other words, the estimated GP value of the Laurdan Frank Condon state was already somewhat relaxed in situations where fast solvent relaxation occurs. Instead, as shown in **Fig. 1e-f**, we estimated the of the GP value of apparent Franck-Condon state (GP FC) from the amplitude and offset of the fitted exponential functions. For our data, a double exponential decay fit was required. The GP FR represents the *ν(∞)* in TRES where the solvent relaxation was fully completed. In our data, GP FR was acquired from the offset of the time resolved GP curve fitting. The difference in total amplitudes of the two respective exponential function $\Delta GP = \sum F_i$, represents the extent of solvent relaxation, known as $\Delta\nu$ as in TRES. The kinetics of solvent relaxation known as τ_r in TRES is represented by the intensity averaged decay constant of the time-resolved GP curve, referred to as Ave GPτ here. The time-resolved GP decay curve was tail fitted to double exponential decay function in the lifetime range of 0.3 ns to 20 ns (**Fig. 1e-f**). Finally, the conventional steady state GP was calculated by summing up collected photons in the blue and green spectral windows, respectively, and is called GP SS in the current study. GP SS is predominantly determined by the amounts of photons transferred from blue to green channel in the given lifetime of excited Laurdan molecule. It is partially influenced by the Franck-Condon spectrum and proportion of excited molecules that manage to relax fast enough before they emit the photon.

Supplementary Figures:

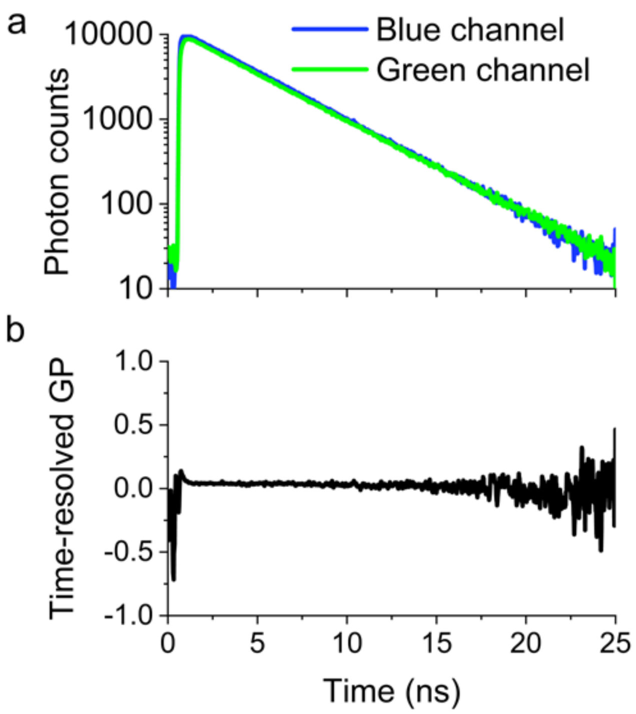

Supplementary Figure 1. Time resolved GP plot of ATTO425. (**a**, **b**) The lifetime decay of ATTO425 in the blue and green channel acquired under the same setting as imaging Laurdan in lipid vesicles (**a**), and the time-resolved GP plot of ATTO425 (**b**). Note that in absence of solvent relaxation, the time resolved GP become a straight line.

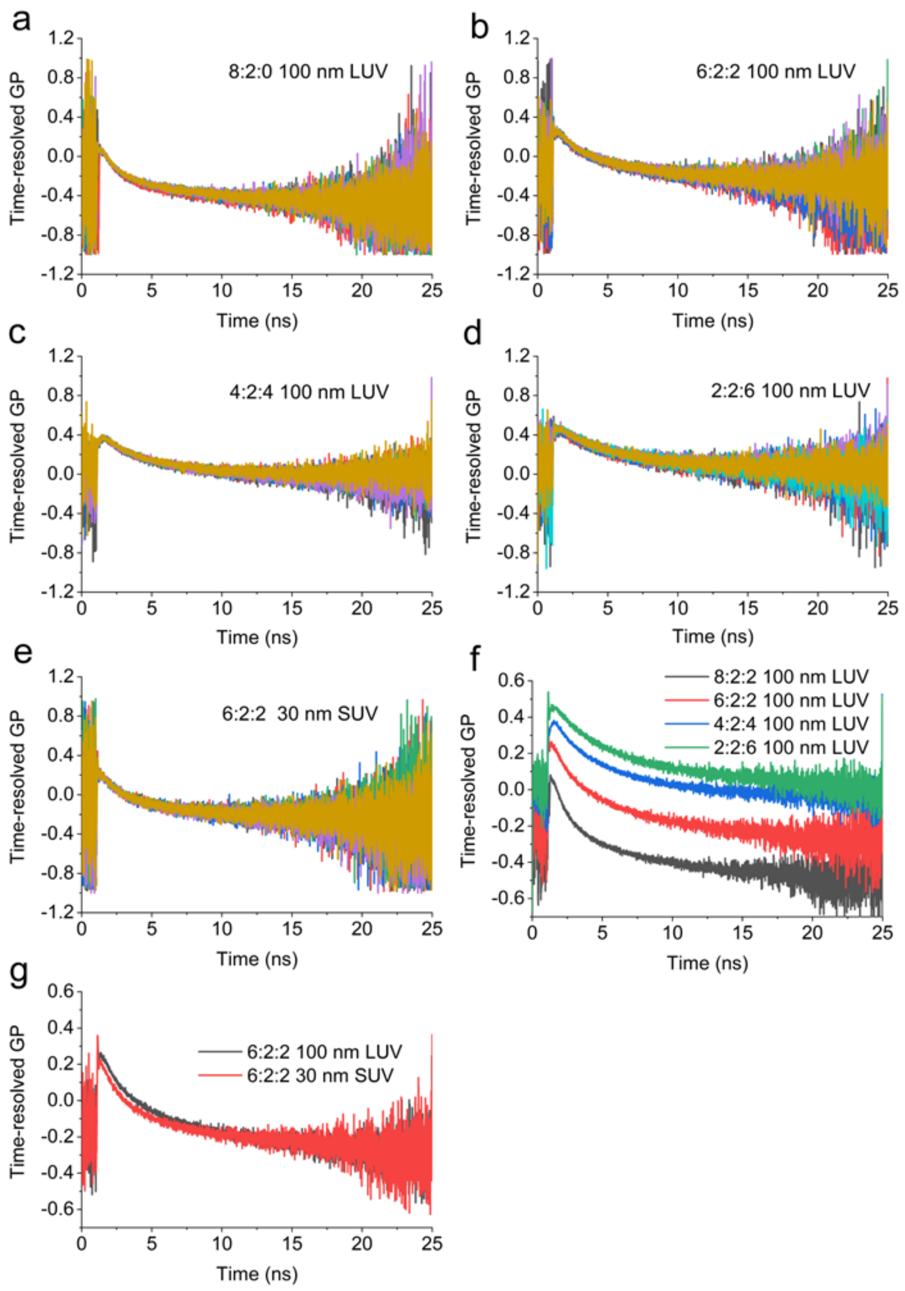

Supplementary Figure 2. Time-resolved GP curves of model membranes with different cholesterol levels and diameter. (**a-e**) Time-resolved GP curves of model membranes with the indicated diameter and ratio of DOPC/sphingomyelin/cholesterol. (**f, g**) Averaged time-resolved GP curves of data shown in a-e (**f**) or b and e (**g**).

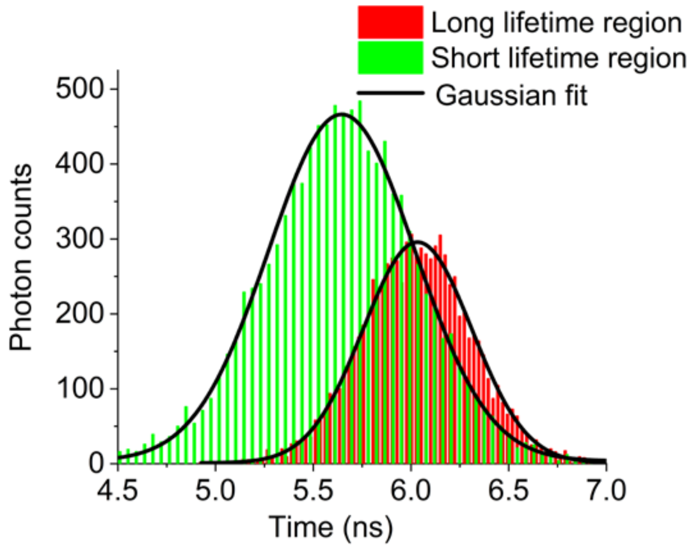

Supplementary Figure 3. Pixel lifetime histograms of the short and long Laurdan lifetime regions of the plasma membrane collected with a SAF objective and shown in Fig. 4b middle and bottom, respectively. The histograms were fitted to single Gaussian distribution functions that peaked at 5.65 ns and 6.03 ns, respectively, and had overall fit of R^2 = 0.995 and R^2 = 0.991 for short and long lifetime regions, respectively.
